# Increased risk for hypothyroidism associated with carbon monoxide poisoning: a nationwide population-based cohort study

**DOI:** 10.1038/s41598-019-52844-9

**Published:** 2019-11-11

**Authors:** Chien-Cheng Huang, Chung-Han Ho, Yi-Chen Chen, Chien-Chin Hsu, Hung-Jung Lin, Shih-Bin Su, Jhi-Joung Wang, How-Ran Guo

**Affiliations:** 1 Department of Emergency Medicine, Chi Mei Medical Center, Tainan, Taiwan; 2 Department of Environmental and Occupational Health, College of Medicine, National Cheng Kung University, Tainan, Taiwan; 3 Department of Senior Services, Southern Taiwan University of Science and Technology, Tainan, Taiwan; 4 Department of Medical Research, Chi Mei Medical Center, Tainan, Taiwan; 5 Department of Pharmacy, Chia Nan University of Pharmacy and Science, Tainan, Taiwan; 6 Department of Biotechnology, Southern Taiwan University of Science and Technology, Tainan, Taiwan; 7 Department of Emergency Medicine, Taipei Medical University, Taipei, Taiwan; 8 Department of Occupational Medicine, Chi Mei Medical Center, Tainan, Taiwan; 9 Department of Leisure, Recreation and Tourism Management, Southern Taiwan University of Science and Technology, Tainan, Taiwan; 10 Allied AI Biomed Center, Southern Taiwan University of Science and Technology, Tainan, Taiwan; 11 Department of Occupational and Environmental Medicine, National Cheng Kung University Hospital, Tainan, Taiwan

**Keywords:** Thyroid diseases, Thyroid diseases

## Abstract

Carbon monoxide poisoning (COP) may cause injuries to the central nervous and endocrine systems, which might increase the risk of developing hypothyroidism. We wanted to evaluate the association between COP and the risk of developing hypothyroidism because epidemiological data on this potential association are limited. We conducted a nationwide population-based cohort study using the Nationwide Poisoning Database and identified 24,328 COP subjects diagnosed between 1999 and 2012. By matching the index date and age, we selected 72,984 non-COP subjects for comparison. Subjects with thyroid diseases and malignancy before 1999 were excluded. We followed up the two groups of subjects until 2013 and compared the risk of developing hypothyroidism. COP subjects had a significantly higher risk for hypothyroidism than non-COP subjects (adjusted hazard ratio [AHR]: 3.8; 95% confidence interval [CI]: 3.2–4.7) after adjusting for age, sex, underlying comorbidities, and monthly income, and the AHR was particular higher in subjects with diabetes mellitus, hyperlipidemia, and mental disorder. The increased risk was highest in the first month after COP (AHR: 41.0; 95% CI: 5.4–310.6), and the impact remained significant even after 4 years. In conclusion, COP was associated with an increased risk for hypothyroidism. Further studies regarding the underlying mechanisms are warranted.

## Introduction

Carbon monoxide poisoning (COP) is an important issue in public health because it is one of the leading causes of poisonings worldwide^1^. There are about 1,000–2,000 accidental deaths due to COP in the US annually, resulting from an estimate of 50,000 exposures each year^1^. In the past 10 years, suicidal COP has increased greatly in certain countries because carbon monoxide (CO) is odorless and ultimately fatal, which is thought to be a perfect tool for committing suicide^2^. In Taiwan, the incidence of suicidal COP by charcoal burning increased from 0.22 to 5.4 per 100,000 people between 1999 and 2009, a nearly 25-fold increase^2^.

Because CO has 250 times higher affinity for hemoglobin than for oxygen, even a small amount of CO can cause severe tissue hypoxia in all the organs, especially the brain and the heart, both of which are organs with the highest oxygen demand^3–5^. In addition to hypoxia, COP induces immunological and inflammatory damages to all the body organs via producing reactive oxygen species, which are longer lasting and cause effects independent of hypoxia^6,7^. Hypoxia and immunological and inflammatory damages may result in various morbidities and even mortality^3–5^. The most commonly known morbidity is brain injury with neurological sequelae, which include difficulty with higher intellectual functions, short-term memory loss, dementia, amnesia, psychosis, irritability, a strange gait, speech disturbances, Parkinson’s disease-like syndromes, cortical blindness, and a depressed mood^6–9^. Thyroid function is regulated by the hypothalamus-pituitary-thyroid (HPT) axis, and therefore an injury to both the brain (i.e. hypothalamus and pituitary gland) and the local organ (i.e. thyroid gland) may contribute to hypothyroidism^10–14^. Because COP leads to hypoxia, which can affect both the brain and the thyroid gland, it is plausible that COP may lead to hypothyroidism. However, we did not find studies on this issue upon searching the PubMed and Google Scholar using the key words “carbon monoxide,” “poisoning,” “intoxication,” “hypothyroidism,” “thyroid,” and “endocrine.” Therefore, we conducted this study to evaluate the association between COP and the risk of developing hypothyroidism.

## Results

The mean age ± standard deviation of subjects in both cohorts was 36.4 ± 15.4 years after matching (Table 1). The age subgroup of 20–34 years had the largest proportion of subjects (39.3%), followed by the 35–49 years subgroup (31.8%). COP subjects had a larger proportion of females (50.6% vs. 49.8%, *p* = 0.045) and a higher prevalence of underlying comorbidities, including hypertension, diabetes mellitus, hyperlipidemia, rheumatoid arthritis, connective tissue disease, drug abuse, and mental disorder, and higher percentage of lower monthly income than the non-COP subjects.

**Table 1:** Demographic characteristics and underlying comorbidities in both COP and non-COP subjects.

Variable	COP subjects n = 24,328	Non-COP subjects n = 72,984	*p*-value
Age (years)	36.4 ± 15.4	36.4 ± 15.4	0.992
**Age (years)**			
<20	2695 (11.1)	8088 (11.1)	>0.999
20–34	9559 (39.3)	28675 (39.3)	
35–49	7739 (31.8)	23217 (31.8)	
50–64	3036 (12.5)	9106 (12.5)	
≥65	1299 (5.3)	3898 (5.3)	
**Sex**			
Female	12303 (50.6)	36368 (49.8)	0.045
Male	12025 (49.4)	36616 (50.2)	
**Underlying comorbidity**			
Hypertension	2807 (11.5)	7390 (10.1)	<0.001
Diabetes mellitus	1460 (6.0)	3382 (4.6)	<0.001
Hyperlipidemia	1978 (8.1)	5108 (7.0)	<0.001
Rheumatoid arthritis	275 (1.1)	548 (0.8)	<0.001
Connective tissue disease	206 (0.9)	451 (0.6)	<0.001
Vitiligo	11 (0.1)	24 (<0.1)	0.380
Scleroderma	1 (<0.1)	3 (<0.1)	>0.999
Psoriasis	185 (0.8)	515 (0.7)	0.381
Drug abuse	1183 (4.9)	717 (1.0)	<0.001
Mental disorder	7785 (32.0)	9866 (13.5)	<0.001
**Monthly income (NTD)**			
<19,999	17550 (72.1)	45327 (62.1)	<0.001
20,000–39,999	5418 (22.3)	20343 (27.9)	
≥40,000	1360 (5.6)	7314 (10.0)	

COP, carbon monoxide poisoning; NTD, new Taiwan dollars. Data are expressed as mean ± standard deviation or n (%).

**Figure 1:**
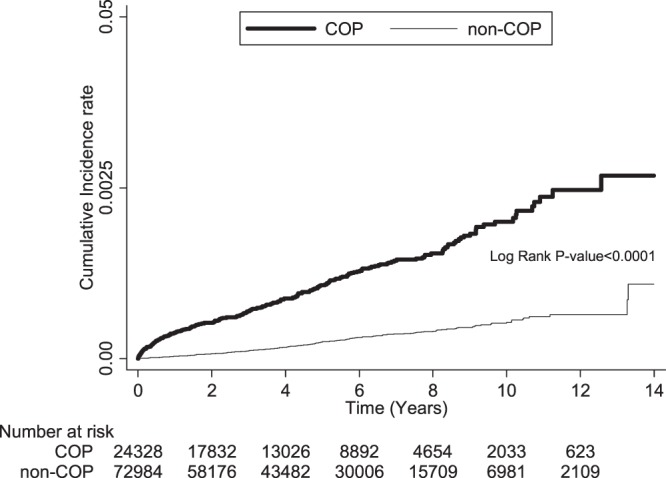
Comparison of the risk of developing hypothyroidism between COP and non-COP subjects by Kaplan–Meier’s method and log-rank test. COP, carbon monoxide poisoning.

In the overall comparison, COP subjects had a significantly higher risk of developing hypothyroidism than non-COP subjects (adjusted hazard ratio [AHR]: 3.8; 95% confidence interval [CI]: 3.2–4.7). The AHR was particularly higher in subjects with diabetes mellitus (AHR: 9.5; 95% CI: 4.2–21.1), hyperlipidemia (AHR: 5.2; 95% CI: 2.9–9.2), and mental disorder (AHR: 5.5; 95% CI: 3.8–8.1) (Table 2). In the subjects without mental disorder, the AHR was 3.2 (95% CI: 2.5−4.1) (Supplemental Table 1). COP subjects also had a higher risk of developing hypothyroidism in the subgroups of rheumatoid arthritis and drug abuse (AHR: 8.2; 95% CI: 0.9–78.5 and AHR: 5.2; 95% CI: 0.7–39.6, respectively), but the increases did not reach statistical significance. The risk of developing hypothyroidism associated with COP was similar between the two sexes (female: AHR: 3.9, 95% CI: 3.1–4.9 vs. male: AHR: 3.2, 95% CI: 2.0–5.1). COP subjects had a significantly higher risks of developing hypothyroidism during the whole follow-up period (Fig. 1; *p* < 0.001 for log-rank tests), even after 4 years (AHR: 2.8; 95% CI: 2.1–3.8). The highest risk was observed in the first month (AHR: 41.0; 95% CI: 5.4–310.6). We evaluated the proportional hazard assumption of the Cox model separately from early to the end of the duration and found the proportional hazard assumption was not violated in every time period. The adjusted competing risks hazard ratio showed the similar results.

**Table 2:** Comparison of the risk for hypothyroidism between COP and non-COP subjects using Cox proportional hazards regression analysis.

Variable	COP subjects n = 24,328			Non-COP subjects n = 72,984			AHR (95% CI)*	AHR^SD^ (95% CI)
	case (%)	PY	rate	case (%)	PY	rate		
Overall analysis	256 (1.1)	118003.2	2.2	191 (0.3)	388039.6	0.5	3.8 (3.2−4.7)	3.5 (2.9−4.2)
**Stratified analysis**								
**Age (years)**								
<20	13 (0.5)	16831.6	0.8	13 (0.2)	51829.3	0.3	2.8 (1.3−6.0)	2.7 (1.3−5.9)
20–34	87 (0.9)	47784.4	1.8	47 (0.2)	154037.0	0.3	4.6 (3.2−6.7)	4.3 (3.0−6.3)
35–49	101 (1.3)	36764.0	2.8	63 (0.3)	123190.5	0.5	4.5 (3.2−6.2)	4.1 (3.0−5.6)
50–64	36 (1.2)	12008.6	3.0	46 (0.5)	41774.1	1.1	2.5 (1.6−3.9)	2.2 (1.4−3.4)
≥65	19 (1.5)	4614.5	4.1	22 (0.6)	17208.8	1.3	3.1 (1.7−5.8)	2.6 (1.4−4.9)
**Sex**								
Female	216 (1.8)	61214.7	3.5	153 (0.4)	195917.2	0.8	3.9 (3.1−4.9)	3.6 (2.9−4.4)
Male	40 (0.3)	56788.5	0.7	38 (0.1)	192122.5	0.2	3.2 (2.0−5.1)	2.8 (1.8−4.4)
**Underlying comorbidity**								
Hypertension	43 (1.5)	10044.9	4.3	36 (0.5)	31541.2	1.1	3.5 (2.2−5.5)	3.0 (2.0−4.7)
Diabetes mellitus	28 (1.9)	4966.4	5.6	8 (0.2)	13774.6	0.6	9.5 (4.2−21.1)	8.4 (3.9−18.2)
Hyperlipidemia	37 (1.9)	6799.9	5.4	20 (0.4)	20122.2	1.0	5.2 (2.9−9.2)	4.7 (2.7−8.2)
Rheumatoid arthritis	5 (1.8)	1040.6	4.8	1 (0.2)	2403.4	0.4	8.2 (0.9−78.5)	7.6 (0.9−66.0)
Connective tissue disease	4 (1.9)	732.5	5.5	3 (0.7)	1761.7	1.7	3.6 (0.7−18.5)	3.2 (0.6−18.4)
Vitiligo	0	—	—	0	—	—	—	
Scleroderma	0	—	—	0	—	—	—	
Psoriasis	0	—	—	1 (0.2)	2075.3	0.5	—	
Drug abuse	15 (1.3)	3869.2	3.9	1 (0.2)	2534.2	0.4	5.2 (0.7−39.6)	4.8 (0.6−36.0)
Mental disorder	137 (1.8)	30038.9	4.6	35 (0.4)	42475.6	0.8	5.5 (3.8−8.1)	4.9 (3.4−7.1)
**Follow-up period**								
<1 month	19 (0.1)	1953.5	9.7	1 (<0.1)	6052.6	0.2	41.0 (5.4−310.6)	39.5 (5.5−285.0)
1–6 months	40 (0.2)	9395.5	4.3	10 (<0.1)	29614.6	0.3	10.5 (5.2−21.4)	10.4 (5.2−20.9)
7–12 months	26 (0.1)	10642.5	2.4	11 (<0.1)	33938.5	0.3	6.4 (3.1−13.3)	6.4 (3.2−12.8)
1–2 years	28 (0.1)	19339.7	1.5	23 (<0.1)	62507.6	0.4	3.1 (1.7−5.5)	3.1 (1.9−5.0)
2–4 years	55 (0.3)	30935.1	1.8	48 (0.1)	101969.5	0.5	3.3 (2.2−4.9)	3.3 (2.2−4.8)
≥4 years	88 (0.7)	45737.0	1.9	98 (0.2)	153957.0	0.6	2.8 (2.1−3.8)	2.8 (2.1−3.7)

COP, carbon monoxide poisoning; HR, hazard ratio; AHR, adjusted hazard ratio; CI, confidence interval; PY, person-year. *Adjusted for sex, hypertension, diabetes mellitus, hyperlipidemia, rheumatoid arthritis, connective tissue disease, vitiligo, scleroderma, psoriasis, drug abuse, mental disorder, and monthly income. AHR^SD^, adjusted competing risks hazard ratio.

Cox proportional hazards regression analysis showed that COP, older age, female sex, hyperlipidemia, mental disorder, and higher monthly income ( ≥ 40,000 New Taiwan Dollars) were independent predictors for hypothyroidism after adjusting for all the other variables in the full model, including age, sex, hypertension, diabetes mellitus, hyperlipidemia, rheumatoid arthritis, connective tissue disease, psoriasis, drug abuse, mental disorder, and monthly income (Table 3). The reduced model revealed that age, sex, hyperlipidemia, and mental disorder were independent predictors for hypothyroidism. In the COP subjects, those who had received hyperbaric oxygen therapy (HBOT) had a significantly higher risk of developing hypothyroidism than non-COP subjects (AHR: 4.0; 95% CI: 2.9−5.5 in the reduced model). While the risk was also higher in the COP subjects with acute respiratory failure (ARF), the increase did not reach statistical significance (AHR: 3.2; 95% CI: 0.4−22.9).

**Table 3:** Independent predictors for hypothyroidism in all the subjects by Cox proportional hazards regression analysis.

Variable	No. of case	Full Model	Reduced Model^#^
	N (%)	AHR (95% CI)^*^	AHR (95% CI)^†^
COP	256 (1.1)	3.8 (3.2−4.7)	3.9 (3.2−4.7)
COP with ARF	1 (0.3)	3.3 (0.5−23.8)	3.2 (0.4−22.9)
COP with HBOT	53 (0.9)	4.1 (3.−5.6)	4.0 (2.9−5.5)
**Age (years)**			
<20	26 (0.2)	1	1
20–34	134 (0.4)	1.7 (1.1−2.6)	1.7 (1.1−2.6)
35–49	164 (0.5)	2.3 (1.5−3.6)	2.4 (1.5−3.6)
50–64	82 (0.7)	3.0 (1.9−4.8)	3.1 (2.0−5.0)
≥65	41 (0.8)	3.4 (2.0−5.8)	3.7 (2.2−6.2)
**Sex**			
Female	369 (0.8)	4.6 (3.6−5.9)	4.6 (3.6−5.9)
Male	78 (0.2)	1	1
**Underlying comorbidity**			
Hypertension	79 (0.8)	1.2 (0.9−1.7)	−
Diabetes mellitus	36 (0.7)	1.0 (0.7−1.5)	−
Hyperlipidemia	57 (0.8)	1.5 (1.0−2.1)	1.5 (1.2−2.2)
Rheumatoid arthritis	6 (0.7)	0.7 (0.3−1.6)	−
Connective tissue disease	7 (1.1)	1.5 (0.7−3.2)	−
Psoriasis	1 (0.1)	0.4 (0.1−2.8)	−
Drug abuse	16 (0.8)	1.3 (0.8−2.2)	−
Mental disorder	172 (1.0)	1.9 (1.6−2.4)	2.0 (1.6−2.5)
**Monthly income (NTD)**			
<19,999	333 (0.5)	1	1
20,000–39,999	74 (0.3)	0.8 (0.5−1.1)	0.7 (0.6−1.0)
≥40,000	40 (0.5)	1.5 (1.1−2.1)	1.5 (1.1−2.1)

COP, carbon monoxide poisoning; HR, hazard ratio; AHR, adjusted hazard ratio; CI, confidence interval; ARF, acute respiratory failure; HBOT, hyperbaric oxygen therapy; NTD, New Taiwan Dollars. ^*^Adjusted for age, sex, hypertension, diabetes mellitus, hyperlipidemia, rheumatoid arthritis, connective tissue disease, psoriasis, drug abuse, mental disorder, and monthly income. ^†^Adjusted for age, sex, hyperlipidemia, mental disorder, and monthly income. ^#^Both the full and reduced models were established among all subjects, and the likelihood ratio test presented similar model effect between full and reduced models (*p* = 0.553).

## Discussion

This nationwide population-based cohort study showed that COP subjects had a significantly higher risk of developing hypothyroidism, especially in those with diabetes mellitus, hyperlipidemia, and mental disorder. The impact of COP on the increased risk of developing hypothyroidism was highest in the first month after COP and remained significant even after 4 years. Older age, female sex, hyperlipidemia, mental disorder, and higher monthly income were also independent predictors for hypothyroidism, in addition to COP.

Injuries to the HPT axis due to COP-related hypoxia and immunological and inflammatory damages may be the causes for hypothyroidism. Hypothyroidism is a common thyroid disorder in the adult population and is more prevalent in older women^15^. The common etiology is through an autoimmune mechanism, such as primary atrophic hypothyroidism, Hashimoto’s thyroiditis, radioactive iodine therapy, or thyroid surgery^15^. The HPT axis is a part of the neuroendocrine system responsible for the regulation of metabolism. While earlier studies in rats reported that exposure to 100 ppm CO induced a decrease in the levels of hypothalamic noradrenaline, brain serotonin, and serum thyroxine^16,17^, human studies on the effects of CO on the hypothalamus, pituitary gland, or thyroid are very limited. In addition to exogenous CO due to poisoning, endogenous CO itself is a neuromodulator that influences the physiological and pathological processes in the central and peripheral nervous systems^18^, which suggests that COP may cause morbidities by affecting hormone regulation in the human body.

We found that the impact of COP on the increased risk of developing hypothyroidism was larger in the subgroups of subjects with diabetes mellitus, hyperlipidemia, and mental disorder. In other words, in the populations with diabetes mellitus, hyperlipidemia, or mental disorder, people with COP had a higher risk for hypothyroidism than those without COP. In the population without mental disorder, the risk for hypothyroidism was still high (AHR: 3.2), which indicates that mental disorder might not have a strong interaction with COP. Because these are novel findings of this study, mechanistic evidences of the associations are limited. Diabetic patients are known to be more vulnerable to hypothyroidism than the general population (6% vs. 3%)^15^. One of the reasons is that the presence of one autoimmune disease may increase the risk for another autoimmune disease^15^. For example, previous studies have reported that up to 30% of females with type 1 diabetes have thyroid disease^15^ and that young patients with type 1 diabetes have a higher risk of developing thyroid disorders^19^. Type 2 diabetes also increases the risk of developing thyroid dysfunction due to insulin resistance, which is caused by perturbed genetic expression of a constellation of genes along with physiological aberrations leading to impaired glucose utilization and disposal in muscles, overproduction of hepatic glucose output, and enhanced absorption of splanchnic glucose^20^. Thyroid function significantly affects lipoprotein metabolism^21^ and mental status^22^. Hypothyroidism is associated with increases in total cholesterol, low-density lipoprotein cholesterol, and triglycerides as well as a decrease in the high-density lipoprotein cholesterol level^21,23^. Thyroid hormones have important actions in the brain, and overt hypothyroidism has major effects on neuropsychiatric function, which may result in mental disorders^22,24^. Treatment of hypothyroidism tends to improve related mental disorders^24^. However, the reasons why patients with hyperlipidemia or mental disorders are vulnerable to hypothyroidism are unclear.

In our study, older age and female sex were independent predictors for hypothyroidism, which is consistent with previous studies^25–28^. A national survey in the US reported that both the thyroid-stimulating hormone level and the presence of antithyroid antibodies are greater in women and increase with age^27^. The Framingham study reported a 4.4% prevalence of hypothyroidism in older participants (age > 60 years) and found that women exhibited hypothyroidism (5.9%) more often than men (2.3%)^26^.

Although this study had the major strengths of nationwide data and casted some light on an unanswered question, there were some limitations. First, details of some variables related to hypothyroidism such as family history were not available in the database. However, we had excluded potential subjects with thyroid diseases or malignancy and included variables of several underlying comorbidities, which may serve as surrogates for the unavailable confounders, and therefore the confounding effect may be minimized. Second, there is no information about the amount, duration, severity, and intent of CO exposure in the Taiwan National Health Insurance Research Database, and therefore the dose-response relationship could not be assessed in this study. Even though the exposure dose is an important factor affecting the prognosis of COP, however, it is hard to determine in clinical settings. In general, except for investigation or research purposes, reconstruction of the exposure dose is rarely done for patients. Therefore, the patient is likely to be the only source of information in many cases, but information such as the concentration and duration of exposure is usually hard to validate, even if the patient is able to provide it. Although COHb is an objective biomarker of exposure, it is hard to estimate the exposure dose accurately on the basis of COHb without accurate information on the duration of exposure, which is often unavailable. Third, this study revealed that COP subjects who received HBOT also had a higher risk for hypothyroidism, with an AHR similar to the overall COP cohort in comparison with the non-COP cohort. Whereas this might imply that HBOT does not have a beneficial effect on the occurrence of hypothyroidism, it needs further studies that make direct comparison between COP subjects with and without HBOT (i.e., 100% normobaric oxygen only) to provide more specific and accurate answers. Fourth, we did not investigate whether or not those who developed hypothyroidism within a few weeks following exposure remained hypothyroid subsequently. Further study is needed to clarify this issue. Fifth, the International Classification of Diseases, Ninth Revision, Clinical Modification (ICD-9-CM) coding made by the emergency physicians might be incorrect and might need validation. However, in our recent hospital-based study^29^, after a chart review we found that all the ICD-9-CM diagnostic coding of COP made in the emergency department were correct. In addition, we used at least one hospitalization or at least three ambulatory care claims to define hypothyroidism and underlying comorbities, which could more confirm the correct diagnosis and avoid possible up-coding. Sixth, although this was a nationwide study, whether it could be generalized to other nations, because of differences in race, culture, and disease treatments, needs to be validated.

## Conclusions

This nationwide population-based cohort study showed that COP significantly increased the risk of developing hypothyroidism, especially in subjects with diabetes mellitus, hyperlipidemia, and mental disorders. The highest risk was observed in the first month after COP, and the increased risk persisted even after 4 years of COP. Possible mechanisms include direct damages by COP to the central nervous system and the thyroid gland via hypoxia and indirect immunological and inflammatory reactions resulted from COP, which result in thyroid dysfunction and then hypothyroidism. Further studies on the detailed mechanisms are needed to validate findings in this epidemiological study.

## Methods

### Data source

The National Poisoning Database (NPD) and the Longitudinal Health Insurance Database 2000 (LHID2000), two sub-databases of the Taiwan National Health Insurance Research Database, were used for this study. Because the Taiwan National Health Insurance program covers nearly 100% of Taiwan’s population, the NPD represents all the poisonings including COP in Taiwan between 1999 and 2013^30^. The National Health Insurance Research Database contains registration files and original claim data for reimbursement^30^. Large, computerized databases derived from this system by the National Health Insurance Administration (the former Bureau of National Health Insurance, BNHI), Ministry of Health and Welfare (the former Department of Health, DOH), Taiwan, and maintained by the National Health Research Institutes, Taiwan, are provided to scientists in Taiwan for research purposes^30^.

### Identification of COP and non-COP subjects

**Figure 2:**
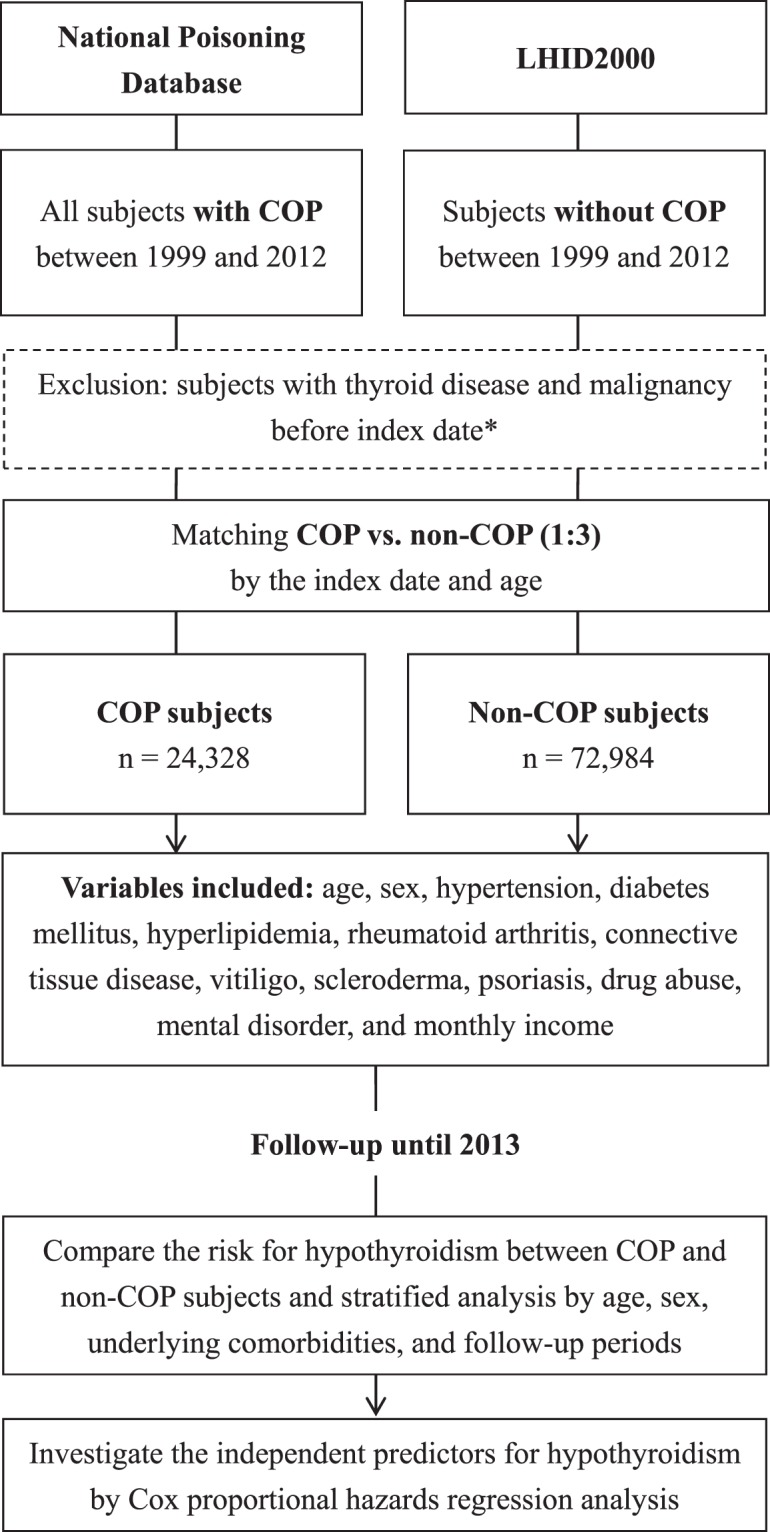
Flow chart of this study. LHID, Longitudinal Health Insurance Database; COP, carbon monoxide poisoning. *Index date: the date of admission or ambulatory care.

We used the NPD to identify all the COP subjects in Taiwan diagnosed between 1999 and 2012 and non-COP subjects from the LHID2000 by matching (1:3) by index date (i.e. date of admission or ambulatory care) and age (Fig. 2). The index date in the non-COP subjects was determined by the index date of the matched COP subjects. We adopted the matching by 1:3 according to previous studies^31,32^. Potential subjects with thyroid diseases and malignancy before the index date were excluded because these two conditions may affect the incidence of hypothyroidism. Thyroid diseases were defined as ICD-9-CM 240–246 or 193. Malignancy was defined as ICD-9-CM 140–208.

### Definitions of included variables

COP was defined as ICD-9-CM codes of 986, E868, E952, or E982 in either hospitalization or emergency department care as one of the main diagnoses^31–34^. Hypothyroidism was defined as having the ICD-9-CM code 244 (i.e., acquired hypothyroidism) as one of the main diagnoses in at least one hospitalization or at least three ambulatory care claims. By this definition, we included only cases of acquired hypothyroidism, and the etiology is either a thyroid disease (primary hypothyroidism) or a hypothalamic-pituitary disease (central hypothyroidism, as in cases with low thyroid stimulating hormone [TSH] and thyroid hormone)^35^. Primary hypothyroidism may be either subclinical (high serum TSH and normal serum free thyroxine [T4] concentrations) or overt (high serum TSH and low serum free T4 concentrations)^35^. For example, patients with low T3 but normal T4 and TSH could present non thyroidal illness syndrome instead of hypothyroidism, and such cases would not be included in our analyses. Likewise, autoimmune thyroid disease (i.e., ICD-9-CM 245) and euthyroid sick syndrome (i.e., ICD-9-CM 790.94) were not included, neither. However, a middle elevated TSH with normal thyroid hormones could indicate resistance to TSH, and the patient would fit our case definition. Age subgroups were classified as < 20, 20–34, 35–49, 50–64, and ≥65 years. Underlying comorbidities were defined as hypertension (ICD-9-CM 401–405), diabetes mellitus (ICD-9-CM 250), hyperlipidemia (ICD-9-CM 272), rheumatoid arthritis (ICD-9-CM 714), connective tissue disease (ICD-9-CM 710), vitiligo (ICD-9-CM 709.01), scleroderma (ICD-9-CM 701.0), psoriasis (ICD-9-CM 696), drug abuse (ICD-9-CM 303–305), and mental disorder (ICD-9-CM 290–302, 306–319). To ensure the accuracy of diagnoses, we included only the cases with the same diagnoses in at least one hospitalization or at least three ambulatory care claims. We included these underlying comorbidities because they were possible confounding factors. ARF was defined as ICD-9-CM: 518.81 or 518.84 or management codes 960, 9601, 9602, 9603, 9604, 9605, 9390, 9391, or 311. HBOT was defined as management codes 47054C, 9395, 59003B, 59004B, 59003A, or 59004A. We included ARF and HBOT in order to investigate the effects of COP severity (ARF stands for a more severe COP) and HBOT on the risk of developing hypothyroidism. Although we did not review the diagnoses in the claims, the National Health Insurance randomly samples claims for reviews by appointed experts on a regular basis.

### Ethics statement

This study was conducted strictly according to the Declaration of Helsinki and approved by the Institutional Review Board at Chi-Mei Medical Center. Informed consent from the subjects is waived because the NPD contains de-identified information, which does not affect the right and welfare of the subjects.

### Data analysis

We compared the risk of developing hypothyroidism between COP and non-COP groups by following up until 2013 (Fig. 1). Independent *t*-test was used for continuous variables and chi-square test was used for categorical variables in the comparison of demographic data, underlying comorbidities, and monthly income between COP and non-COP subjects. Cox proportional hazards regression analysis and adjusted competing risks hazard ratio were used for comparing the risk of developing hypothyroidism between COP and non-COP subjects, with adjustment for age, sex, selected comorbidities, and monthly income. Because age, sex, and underlying comorbidities may affect the association between COP and hypothyroidism, we performed stratified analyses to evaluate the possible effect modifications. Stratified analyses by follow-up period were also performed to evaluate whether the effects of COP change over time. Kaplan–Meier method and the log-rank test were used to compare the risk for hypothyroidism between COP and non-COP subjects during the follow-up period. We treated death as a censoring event. In addition, subjects who were out of the National Health Insurance Database during study duration were defined as lost to follow up censoring. Nonetheless, as the insurance is compulsory with a long grace period for premium payment, almost all such cases are deaths with delayed reporting, which are extremely rare.

Multi-variate Cox proportional hazards models were constructed to identify the independent predictors for hypothyroidism and evaluate their effects. A full model was first constructed to provide a comprehensive assessment, which included all the variables selected from uni-variate analyses that are supported by the literature as risk factors for hypothyroidism. To obtain a reduced model that is more useful in clinical setting, we first excluded comorbidities with prevalence less than 1% and then exclude variables that did not reach statistical significance. We used SAS 9.4 for Windows (SAS Institute, Cary, NC, USA) for all analyses. The significance level was set at 0.05 (two-tailed).

## Supplementary information

Supplemental Table 1. The risk of hypothyroidism between COP and non-COP subjects stratified by mental disorder
