# Supplementary material for: Increased risk for hypothyroidism associated with carbon monoxide poisoning: a nationwide population-based cohort study
Source: Sci Rep. 2019 Nov 11;9:16512. doi: 10.1038/s41598-019-52844-9 (PMC6848088; doi:10.1038/s41598-019-52844-9)
Supplement: Supplementary file 1 — Supplemental Table 1. The risk of hypothyroidism between COP and non-COP subjects stratified by mental disorder [file 41598_2019_52844_MOESM1_ESM.docx]

**Increased risk for hypothyroidism associated with carbon monoxide poisoning: a nationwide population-based cohort study**

Chien-Cheng Huang, MD, PhD^1,2,3^, Chung-Han Ho, PhD^4,5^, Yi-Chen Chen, MS^4^, Chien-Chin Hsu, MD, PhD^1,6^, Hung-Jung Lin, MD, MBA^1,6,7^, Shih-Bin Su, MD, PhD^8,9^, Jhi-Joung Wang, MD, PhD^4,10^, How-Ran Guo, MD, MPH, ScD^2,11^

**Supplemental Table 1.** The risk of hypothyroidism between COP and non-COP subjects stratified by mental disorder

| Variable | AHR (95% CI)* | *p*-value |
| --- | --- | --- |
| With mental disorder | 5.5 (3.8−8.1) | <0.001 |
| Without mental disorder | 3.2 (2.5−4.1) | <0.001 |

*Adjusted for sex, hypertension, diabetes mellitus, hyperlipidemia, rheumatoid arthritis, connective tissue disease, vitiligo, scleroderma, psoriasis, drug abuse, and monthly income

**Supplemental Table 2.** The results of proportional test in every time period

| Follow-up period | Rho | P-value |
| --- | --- | --- |
| < 1 month | 0.1265 | 0.5716 |
| 1-6 months | -0.0453 | 0.7487 |
| 7-12 months | -0.1063 | 0.5182 |
| 1-2 years | -0.2156 | 0.1238 |
| 2-4 years | -0.0294 | 0.7656 |
| ≥ 4 years | 0.0424 | 0.5639 |
